# AVATAR Virtual Reality Social therapy (AVATAR_VRSocial) for distressing voices and their interference in social everyday life in early psychosis: protocol of a single-blind parallel group randomised controlled feasibility study

**DOI:** 10.1136/bmjopen-2024-098004

**Published:** 2025-04-17

**Authors:** Mar Rus-Calafell, Melina Luker, Maren Marzinzik, Phuong-Mi Nguyen, Silvia Schneider, Tobias Teismann, Nils Ehrbar, Ekincan Tas, Xiao Chi Zhang, Clementine Edwards, Mark Huckvale, Tom K J Craig, Philippa Garety, Thomas Ward

**Affiliations:** 1 Mental Health Research and Treatment Centre, Faculty of Psychology, Ruhr-Universität Bochum, Bochum, Germany; 2 German Center for Mental Health, Partner Site Bochum/Marburg, Bochum, Germany; 3 Department of Psychology, King’s College London Institute of Psychiatry Psychology & Neuroscience, London, UK; 4 Avatar Therapy Ltd, London, UK; 5 Department of Health Service and Population Research, King’s College London Institute of Psychiatry Psychology & Neuroscience, London, UK

**Keywords:** Schizophrenia & psychotic disorders, Digital Technology, Psychosocial Intervention, Virtual Reality

## Abstract

**Introduction:**

Around 70% of people with psychosis experience auditory verbal hallucinations (AVHs), which can cause distress and impair the social functioning of the individual. AVATAR therapy works by facilitating a ‘face-to-face’ dialogue between the person and a digital representation (avatar) of their persecutory voice. Although there is cumulative evidence of this way of working with voices, enhancing the therapeutic focus on improved confidence and sense of control of the voices in social situations represents a promising way to boost generalisation of therapy gains into social contexts. We aim to enhance AVATAR therapy by incorporating immersive Virtual Reality (VR) social environments aiming to help the person to deal better with their voices in daily situations.

**Methods and analysis:**

A randomised controlled feasibility trial will be conducted. 40 patients aged 18 or above who are at early stages of psychosis (first episode of psychosis in the last five years) and report distressing and interfering voices will be recruited. Participants will be randomised to receive either a novel, enhanced version of AVATAR therapy (AVATAR_VRSocial) in addition to usual care or usual care alone. Assessor-blinded assessments will be conducted at baseline, 3 months (post-intervention) and 6 months (follow­-up). Key therapeutic targets of AVATAR_VRSocial will be those established by the previous evidence of this approach (ie, power and control, self-esteem and future focus), while introducing exposure and management of distressing voices during social interactions. Analyses will focus on feasibility outcomes (recruitment, retention and completion rates) and preliminary estimates of intervention effects. Qualitative interviews will be carried out with participants allocated to AVATAR_VRSocial to gain a comprehensive understanding of participants’ views on the acceptability of the intervention and research procedures. Thematic analysis of the qualitative interviews will assess the acceptability of the intervention, trial procedures and the new VR technology and software involved.

**Ethics and dissemination:**

The study has received ethical approval from the Ethics Commission at the Faculty of Psychology (Ruhr-Universität Bochum), and there is an independent Trial Steering Committee and Lived Experience Advisory Panel also supporting it. Findings will be disseminated through peer­-reviewed publications, conference presentations and science dissemination events.

**Trial registration number:**

ISRCTN35980117.

**STRENGTHS AND LIMITATIONS OF THIS STUDY**

- AVATAR_VRSocial represents the first attempt to enhance AVATAR therapy for distressing voices with the incorporation of innovative Virtual Reality technology to improve voice interference during daily life.
- The trial is the first to use this cognitive-relational approach and specifically focusing on distressing voices in people at early stages of psychosis (first episode of psychosis<5 years).
- The study uses mixed methods (clinical assessments, questionnaires, qualitative interviews) to respond to the research questions.
- Participants cannot be blinded to the treatment allocation, which could constitute a bias of the data.
- The control condition is *treatment as usual*, so it will not be possible to determine which elements of AVATAR_VRSocial may produce change.

## Background

 Auditory verbal hallucinations (AVHs) or *hearing voices* is the most prevalent form of hallucinations.^1 2^ Between 69% and 82% of individuals with schizophrenia spectrum disorders report experiencing them.^3^ The experience of negative AVH is not only a source of considerable emotional distress but has a significant impact on daily functioning and quality of life.^4^ Deterioration of social functioning manifests in avoidance and withdrawal from social interactions, both main characteristics of social anxiety. This is among the most prevalent and debilitating affective disturbances manifesting in people with psychosis, affecting around 25% of people with first episode of psychosis (FEP)^5^ and seriously hindering recovery.^6^ Particularly for those at early stages of psychosis, the stress associated with stigma^7^ and the distressing nature of the voices themselves—such as conveying a sense of danger—can lead individuals who hear voices to isolate themselves. Considering that such voices often belittle, intimidate and threaten voice-hearers, those affected face unique challenges in their everyday lives. These can range from difficulties disengaging from the voices, a fear of people’s perceptions should they react to the voices in public, struggling to concentrate on conversations with others or working out who is talking, to voices and their content triggering avoidance.^8 9^

While the current therapeutic gold standard for distressing voices, cognitive behavioural therapy for psychosis (CBTp), has shown small to moderate effects,^10 11^ studies suggest only around 50% of people with the disorder benefit from it,^12^ with very limited improvements in treatment response for AVH in recent years.^13^ CBTp is also lengthy and not widely accessible to many in need.^14^ AVATAR therapy is an innovative therapy approach designed to support voice hearers to develop an increased sense of power and control over their voices, through a series of dialogues between the person and a digital representation of the voice (or avatar). It was originally created by Julian Leff in 2013^15^ in the UK. Preliminary evidence for its efficacy was demonstrated through two independent pilot studies^16^,^15^ and further supported by the findings of a large fully powered randomised controlled trial (AVATAR1) comparing AVATAR therapy and supportive counselling.^17^ This study showed a post-therapy effect size of d=0.8 of AVATAR therapy on the primary outcome (total score on the Psychotic Symptom Rating Scales, auditory hallucinations subscale).^18^ Most recently, a multicentre randomised controlled study (AVATAR2) set out to test two versions of the therapy (a brief and extended form) against treatment as usual (TAU) with the aim to support optimisation. The study not only lends additional support to the efficacy of AVATAR therapy compared with TAU, but also shows that an abbreviated form of the therapy can yield similar clinical improvements in both voice distress and voice severity compared with a longer intervention.^19^ However, only AVATAR extended produced a significant reduction in voice frequency in this trial. Although effects on social functioning were not reported in the AVATAR1 RCT^17^ due to lack of suitable validated measures included within the assessment battery, participants who took part in the embedded qualitative study reported an increased level of engagement in social situations and improved confidence around others.^20^ Enhancing the therapeutic focus on improved confidence and sense of control of the voices in social situations represents a promising way to boost generalisation of AVATAR therapy gains into social contexts and reduce anxious avoidance and threat cognitions (including perceived threat from the voices).

Virtual Reality (VR) has long been used to facilitate exposure to feared stimuli mostly for anxiety disorders, with encouraging results for the treatment of psychosis.^21 22^ Virtual environments evoke responses in a participant that match those occurring in the natural environment. This offers a unique opportunity to access the user’s real-time behaviour, including interaction with virtual agents, and allows the person to test out new responses, for example, targeting the safety behaviours and paranoid attributions that can maintain voice-related distress.^23^ Relevant environments, providing emotionally significant content targeting specific emotions and related cognition for a specific population, and optimal induced levels of presence are required when delivering VR-based therapy. Following this evidence, we present a new augmented version AVATAR therapy which includes three additional sessions using VR environments to improve the generalisation of therapy gains to the person’s social functioning.

## Objectives

The primary objective is to assess the feasibility and acceptability of a targeted intervention to improve voices related distress, and social avoidance and distress in people at early stages of psychosis (FEP<5 years^24^). The second main research objective is to convene evidence on clinical outcomes to provide first estimates of efficacy of this enhanced version of AVATAR therapy (AVATAR_VRSocial) in a sample of German individuals with early psychosis, which can inform subsequent development and testing of the therapy. Table 1 summarises the objectives of the study and includes the associated feasibility markers and assessment measures.

**Table 1:** Summary of objectives and related assessment measures

	Objectives	Measures
Primary	Assessing feasibility and acceptability of AVATAR_VR psychotherapy for people who experience distressing voice hearing	Number of patients referred to the study, consented, declined and retainedCompletion rates of each assessment measure and retention at follow-up assessmentsNumber and proportion of consenting participants within AVATAR_VRSocial condition who reach the point of therapy ‘exposure’ (attended at least five of nine therapy sessions)Number of adverse serious and non-serious adverse events in each condition
Secondary	Clinical information about preliminary estimates of clinical effectiveness of AVATAR_VRSocial in addition to treatment as usual vs treatment as usual alone in people in early stages of psychosis	Voice distress, frequency and severity of voices: PSYRATS-AHSocial anxiety and avoidance: O-AS, O-CDQOther voices measures: BAVQ-R; VPDS; Brief assessment of negative hallucinations when outsidePsychiatric symptoms: PSYRATS-DEL, DASS-21. CAINS, Rosenberg-Self-EsteemQuality of life: EQ-5D-5L
Qualitative	Assessing acceptability	AFIMFeedback from Qualitative Interview

AFIM, Acceptability and Feasibility of Intervention Measure; BAVQ-R, Beliefs About Voices Revised; CAINS, Clinical Assessment Interview for Negative Symptoms; DASS21, Depression Anxiety and Stress Scales; EQ-5D-5L, EuroQol BIOMED Research Programme; O-AS, Oxford—Agoraphobic Avoidance Scale; O-CDQ, Oxford Cognitions and Defences Questionnaire; PSYRATS-AH, Psychotic Symptoms Rating Scale—Auditory Hallucinations; PSYRATS-DEL, distress and frequency factors, and total scores on the delusional beliefs subscale of the Psychotic Symptoms Rating Scales; VPDS, Voice Power Differential Scale.

The hypotheses related to clinical outcomes are as follows:

- Compared with TAU, AVATAR_VRSocial therapy added to usual care will reduce distress associated with voices (at post-treatment).
- Compared with TAU, AVATAR_VRSocial therapy added to usual care will reduce social avoidance and social distress (at post-treatment).
- Compared with TAU, AVATAR_VRSocial therapy added to usual care will reduce total voice severity, reduce psychiatric symptoms (depression, anxiety, negative symptoms), improve self-esteem and well-being, and enhance quality of life (at post-treatment).
- Treatment effects will be maintained at follow-up.

## Methods and analysis

### Trial design and flow chart

**Figure 1:**
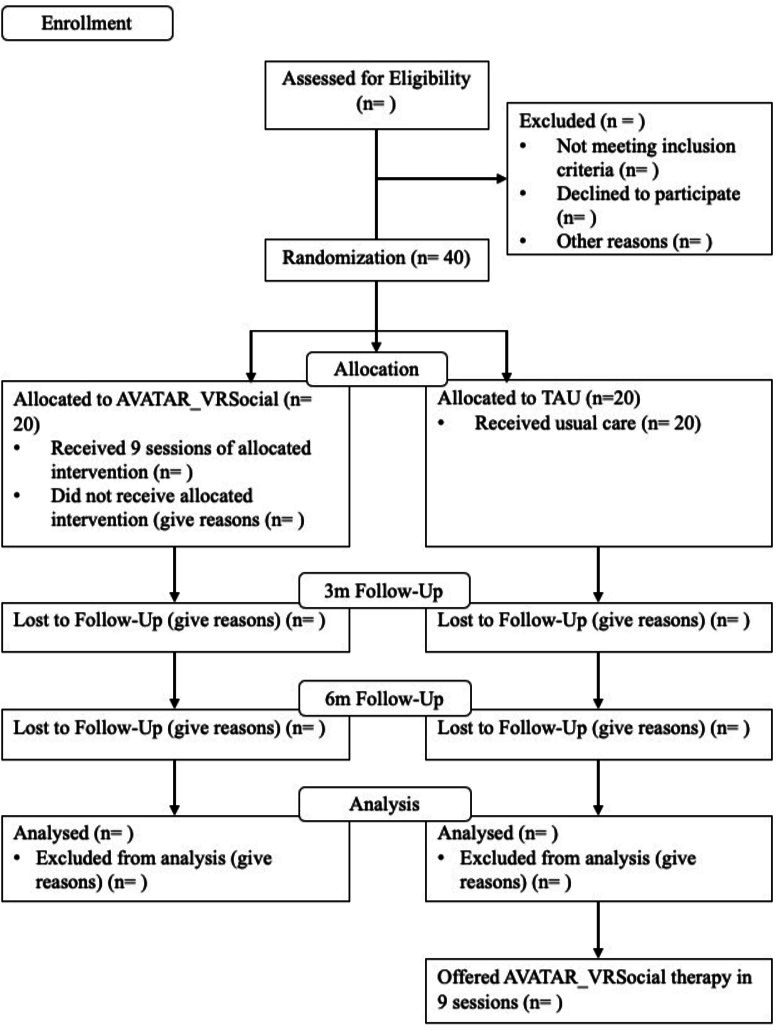
Trial flow chart. TAU, treatment as usual.

The design is a prospective parallel-group feasibility randomised controlled trial with single-blind assessment aiming to evaluate a novel targeted psychological intervention for distressing voices in early psychosis (AVATAR_VRSocial) in addition to usual care versus usual care alone. Assessments will be carried out at baseline (T1), at post-treatment at 3 months (T2) and at 6 months follow-up (T3). The assessments will be carried out by a researcher blinded to treatment allocation. This study will follow active consent. Participants will also be asked for consent at baseline (1) if they consent to be contacted for follow-ups to monitor changes and/or persistence of experiences and (2) if they consent to be contacted to take part in future research linked to the study and its results. Same procedure will be followed at each time point (T2, T3). Figure 1 depicts a summary of the study design.

### Randomisation and blinding

Eligible participants will be randomised after consenting to the study and completion of the baseline assessment. Allocation will follow 1:1 block randomisation, carried out by the study coordinator, using a validated online tool (https://www.sealedenvelope.com/). The study coordinator will be unblinded to treatment allocation and will inform participants of the outcome of the randomisation. Research workers who lead the baseline and follow-up assessments will be blinded to group allocation, but participants and therapists delivering the therapy will not be (it is not possible to blind them to whether the therapy is delivered or received). Participants will be reminded not to discuss their allocation with research workers when they meet them for the assessment. Breaches in blindness will be monitored and recorded and where operationally feasible assessments will be allocated to another (blinded) research worker.

### Participants

The study participants will be 40 people (aged 16 and above) with distressing voices and at early stages of psychosis (FEP 5 years). All research activities (including coordination of recruitment) will be based at the Mental Health Research and Treatment Centre (MHRCT), Ruhr-Universität Bochum, located in North Rhine-Westphalia region (Germany). The primary recruitment pathway will be referrals by medical professionals. Medical service centres, outpatient departments and private practitioners will be contacted for the purpose of recruitment and will act as patient identification centres. Potential participants whose mental health practitioner confirms that they experience distressing auditory hallucinations will be contacted by a member of their clinical team inviting them to learn more about the study. If a participant indicates interest, they will be contacted by a research worker who will confirm eligibility criteria and obtain consent to participate in the research. Potential participants will be offered the opportunity to discuss the study and at least 24 hours to decide whether to participate. The research worker will also assess capacity to provide consent to participate. Informative flyers will also be distributed in practices, which contain information for potential participants. Although the flyer asks participants to approach the research staff via their clinical team, previous experience shows that some will make a direct approach.^17 25^ Further self-referrals are possible because of interest generated through media/public engagement events or through the study website.

#### Inclusion criteria

- Have current frequent and distressing voices (as measured by a score of at least one on each intensity of distress and frequency items of the Psychotic Symptoms Rating Scale—Auditory Hallucinations (PSYRATS-AH)), persisting for at least 6 months and speaking German.
- Confirmed diagnosis of affective or non-affective psychosis (F20-29, F32/33.3 and F31.2 in ICD-10^26^) on the medical records or through consultation with the study clinical team.
- With the onset of the first psychotic episode or first presentation to mental health services in the last five years.
- ≥16 years old.
- Adequate German language skills to provide informed consent and engage with assessment and therapy sessions.
- To be able to give informed consent.

#### Exclusion criteria

- Primary diagnosis of substance disorder, personality disorder or learning disability.
- AVHs secondary to an organic disorder.
- Currently participating or being confirmed to participate in another interventional study in which they are receiving an intervention which uses psychological therapy and focuses on voices.
- Profound visual/hearing impairment or insufficient comprehension of German to be able to engage in assessment or therapy.
- Currently experiencing an acute mental health crisis (ie, being at immediate and serious risk to self or others).

Participants have the right to withdraw from the study at any time for any reason. All efforts will be made to report reasons for withdrawal. If they wish to withdraw from the therapy only, all efforts will be made to obtain follow-up data. The chief investigator (CI) also has the right to withdraw patients from the study in the event of clinical contraindications: the intervention may be discontinued if the participant requests this or if the clinician judges, in consultation with the wider supervisory team, that the intervention is associated with a significant worsening of mental health. In this case, an adverse event (AE) form would also be completed. It is understood by all involved that an excessive rate of withdrawals can render the study uninterpretable; therefore, unnecessary withdrawal of participants should be avoided. Should a participant decide to withdraw from the study, all efforts will be made to report the reason for withdrawal as thoroughly as possible. Should a participant withdraw from therapy only but not from the study, efforts will be made to continue to obtain follow-up data and assess reasons for dropping out, with the permission of the patient.

### Assessments

The assessment of eligibility and confirmation of the inclusion and exclusion criteria will be supported by the use of the Structured Clinical Interview for DSM-V (SCID-5)^1^ and the distress and frequency items of the PSYRATS-AH.^18^

General demographic and relevant clinical data (including age, gender, ethnicity, sexual orientation, employment status, clinical diagnosis, current medication) will be obtained at baseline. Main clinical outcomes, including psychotic and affective symptoms, social anxiety and functioning, quality of life and self-esteem, will be assessed at all three time points (baseline assessment at 0 months, follow-up assessments at 3 months and 6 months).

Psychotic symptoms will be assessed using clinical interview measures, specifically the Psychiatric Symptom Rating Scales for both Auditory Hallucinations (PSYRATS-AH) and Delusions (PSYRATS-DEL),^18^ the Scales for the Assessment of Positive Symptoms^27^ and the Clinical Assessment Interview for Negative Symptoms.^28^ Characteristics of (Voice Characterisation Checklist)^29^ and beliefs about the voice (Beliefs about Voices Questionnaire Revised),^30^ Voice Power Differential Scale (VPDS),^31^ as well as related experiences and coping strategies (Experiencing voices outdoors)^32^ will further be assessed via self-report. Levels of anxiety around leaving the house as well as subsequent avoidance and coping will be assessed using the Oxford Cognitions and Defences Questionnaire^33^ and the Oxford Agoraphobic Avoidance Scale (O-AS).^34^

Depressive symptomatology will be obtained via the Depression, Anxiety and Stress Scale.^35^ Self-esteem will be measured using the Rosenberg Self-Esteem Scale.^36^ An overview of potential traumatic events will be obtained via the Mini-TALE.^37^ The EQ-5D- 5 L^38^ will quantify health-related quality of life.

Participants allocated to the AVATAR_VRSocial Therapy condition will further be asked to complete items regarding anxiety, perceived hostility, sense of presence and sensed presence at the end of each interaction with the avatar (adapted from refs.^39 40^). Following each VR session, participants will be asked to complete a short version of the State Social Paranoia Scale.^41^ Voice activity and content in the past week is monitored at the beginning of each session using select items of the PSYRATS-AH^18^ and the VPDS.^31^ After half of the sessions, participants will be asked to complete a measure of the therapeutic alliance (Working Alliance Inventory-Short Revised).^42^ Before starting the VR sessions, participants will be asked to complete the questionnaires related to social avoidance and social distress. After completion of the final session, participants will rate the acceptability and appropriateness of the therapy via the Acceptability of Intervention Measure and the Intervention Appropriateness Measure.^43^

A qualitative interview will be conducted with a subsample of consecutively recruited participants (n=10). This interview will take place once they have concluded their participation in all assessments in the trial. This interview will ask them for feedback on how they found the therapy, taking part in the trial and the technology/software involved. The interview will be conducted by a research worker or by a member of the Live Experience Advisory Panel with support from the research team.

Assessments and therapy sessions will be audio-recorded (after first establishing consent) to allow for assessment of adherence to the research protocol and assessment ratings. These will be stored securely on password-protected devices and then uploaded to the secure university server. Throughout the recruitment and research process, all efforts will be made to tailor to participants’ needs and preferences. Participants in the study will be reimbursed with a voucher worth €30 in online shopping portals for each baseline, post-therapy and follow-up assessments.

### Trial interventions

#### AVATAR_VRSocial

AVATAR_VRSocial is an augmented version of the AVATAR therapy (as delivered in Craig *et al*^17^) by adding three additional sessions supported by immersive VR technology. Therefore, this therapy consists of a total of 10 sessions (see table 2).

**Table 2:** Summary of the sessions including in AVATAR_VRSocial therapy

	Short description	Number of sessions
AVATAR therapy	Three-way dialogue between participant, avatar and therapist	Seven sessions (six sessions of + one assessment and avatar creation session)
Virtual Reality social scenarios	Interaction with social agents during quotidian situations+personalised negative voice	Three sessions

AVATAR therapy works by facilitating a novel therapeutic context in which a ‘face-to-face’ dialogue between the person and a computerised representation of their voice is facilitated by a therapist. Bespoke software transforms the therapist’s voice to match the pitch and tone of the chosen voice, and the person creates a visual representation of their voice. The voice and image are combined to produce the ‘avatar’ (ie, an animated talking virtual agent with enhanced lip-sync speech and eye blinking), through which the therapist interacts with the voice-hearer, in a three-way dialogue (for further details on the software, please see https://www.avatartherapy.co.uk/). Using this platform, the therapist can speak to the client in their normal voice or in the chosen avatar voice which is a modified version of their normal voice. Where there are multiple voices, the participant selects one to work with (usually the most dominant, frequent and distressing). The embodiment of the voice is enhanced using direct verbatim speech, and enactment of the ascribed character and background of the voice. A comprehensive account of therapeutic targets of AVATAR therapy showed that some therapeutic targets were clearly present for all therapy completers (power and control, self-esteem and future focus), whereas others (eg, maintenance processes, working with trauma, experiential disengagement) were identified in some but not all participants, in line with the tailoring of the intervention to an individualised formulation.^44^

AVATAR therapy as delivered within the pilot work and AVATAR1 trial consists of a total of six sessions (in addition to an initial clinical assessment session including avatar creation) and comprises two phases. In the first phase (Exposure and Assertiveness), the avatar delivers verbatim voice content (including threats and abuse) and the person practices assertive responding. Over time the avatar becomes less hostile as the person develops increased power and control within the dialogue. This cues a second phase with formulation-driven therapeutic targets, which can include work on beliefs about voices, self-concept and trauma. Each AVATAR therapy session consists of three parts: pre-dialogue, active dialogue and post-dialogue debrief. The pre-dialogue and post-dialogue are important for cueing and consolidating key change processes occurring in the dialogue with the avatar. The active dialogue duration is approximately 5 min in early sessions, increasing to 10–15 min in later ones. Participant and therapist are in separated rooms but in constant communication for this part of the session.

For AVATAR_VRSocial, three social virtual scenarios have been created to facilitate generalisation of AVATAR therapy learnings into management of the voice hearing experience during social interactions. The scenarios include (1) taking a bus ride in a full bus, (2) looking for specific groceries in a full supermarket and (3) talking to the receptionist at a doctor’s practice. Each scenario has a specific task to be accomplished by the person. Some responses from the virtual agents are automated and modulated by the person’s verbal response. During the social interaction, the person experiences their personalised voice (ie, the transformed voice matching the voice that distresses them). The aim is to practise taking control of the voices by training what they learnt during the dialogues (eg, disengaging from voices, postponing the conversation) as well as refocusing on the social interactions. The therapist can see and hear everything that is occurring in the VR environment as well as the participant’s responses. Mirroring the avatar dialogues, they will be able to communicate with the participant directly within the VR environment using their own voice, offering support during the interactions. The VR sessions are delivered over three individual sessions assisted by these VR environments.

The virtual scenarios are presented using a wireless head-mounted display (Pico 4) with a resolution of 2160 × 2160 pixels per eye, 122.16° diagonal field of view and mounted headphones. The personalised negative voice is exported from the AVATAR therapy platform to the VR scenarios. The therapist can trigger the negative voice at random intervals (determined by the system) or at his/her own demand depending on the person’s performance. As per the AVATAR therapy sessions, the three VR sessions consisted of three parts: the pre-VR discussion with the therapist, the immersive part using the VR headset (lasting approximately 10 min) and the post-VR debrief with the therapist. The person can request to stop the immersive experience at any point.

At the end of the final session, participants will be asked to complete a self-reported questionnaire to assess the acceptability of the intervention.^43^ The number of active sessions attended (ie, sessions involving active avatar dialogue or VR practice) by the participant will be measured to assess treatment adherence. Fidelity to the clinical manual will be assessed by the therapist by completing a session-by-session checklist of specified targets (these will also be used during training and ongoing supervision; see above). Each session will be audio-recorded with consent. Therapist competence, after completion of training, will be assessed by an expert in AVATAR therapy for general/clinical and avatar-specific skills using ratings adapted from the first AVATAR therapy trial and allowing for differing skill requirements for each level of therapy.

#### Treatment as usual

Participants who are allocated to the TAU arm will continue to receive their usual care. TAU for the participants in this trial will typically consist of medication management, supportive brief counselling sessions and various types of psychosocial support (eg, social work guided support, peer support) and monitoring provided by mental health services, with individual and family psychological therapies offered occasionally. TAU will vary across individuals and clinical teams. Efforts will be put into collecting detailed data on TAU.

Participation will not alter usual treatment decisions about medication and additional psychological and psychosocial interventions, which remain the responsibility of the clinical team. The antipsychotic medication prescribed to participants in the study and psychological and psychosocial interventions provided will be recorded.

### Adverse events

The occurrence of AEs will be monitored actively and systematically, following guidance from the Consolidated Standards of Reporting Trials (CONSORT)^45^ with the extension for social and psychological interventions, and the extension for reporting of harms. AEs are defined as any untoward medical occurrence, unintended disease or injury, or untoward clinical signs in service user/patient participants, whether or not related to the therapy or device which require additional support or input from health professionals. AEs will be initially assessed at three levels of intensity: mild, moderate and severe, which reflect the impact of the event on the person at the time. Please note there is a distinction between ‘severe’ and ‘serious’. Seriousness is the criteria for defining regulatory reporting obligations and the following will be considered as serious adverse events (SAE, categories A–C): all deaths (category A), incidents which acutely jeopardise the health or psychological well-being of the individual, resulting in immediate hospital admission and/or permanent disability (category B) or resulting in injury requiring immediate medical attention (category C).

In any case of an SAE, the CI must be notified within one working day by a member of the trial team becoming aware of the event. The CI (or a clinically qualified delegate) will review all SAE reports received. The CI will keep investigators informed of any safety issues that arise during the course of the trial. The CI is responsible for reporting fatal and life-threatening serious adverse reactions to the competent authorities within 7 days of the CI becoming aware of the event. The TSC will be informed of serious adverse reactions periodically. The chair of the TSC will determine the relatedness of the event to the research assessment, therapy or device based on a temporal relationship, and consideration of whether there were insufficient or inadequate instructions for use, deployment, installation or operation, or any malfunction of the AVATAR software. An AE may also be device-related if it was the result of user error or intentional misuse of AVATAR therapy software. As detailed in the risk analysis conducted for the CE-marking of AVATAR therapy software, we do not anticipate any serious adverse device effects. We do not anticipate any serious therapy or assessment-related AEs; this expectation is based on the safety data from the previous AVATAR trials.^17 19^ Reasons for withdrawal from the study will be recorded. For the final reports of the trial, the numbers, types and severity of AEs by trial condition, as well as discontinuations, will be reported using descriptive statistics.

### Data handling

The CI will act as custodian for the trial data. Data handling is informed by guidelines from the *Deutsche Gesellschaft für Psychologie* (German Psychological Society), the subject-specific guidelines (‘Data Management in Psychological Science’)^46^ and the European Union General Data Protection Regulation. Participants’ data will be pseudoanonymised and stored on a password-protected computer. All trial data will be stored in line with the Data Protection Act and archived in line with sponsor requirements. Data from the assessments will be entered into a central record by research workers using a secure network connection. Audio files named with a unique participant identifier will be stored as computer files on secure RUB servers. Data management will be supported by the trial statistician from MHRTC and Bochum staff and resources (ie, computerised recall centre facility). Additionally, the data will be stored on archive servers of the department of IT Services of RUB. Data will be stored for at least 10 years, and the data protection officer of RUB will supervise the storage of the data and ensure the privacy protection of the individual data subsets. Issues relating to confidentiality will be addressed and potential participants will be advised of the limits of confidentiality (ie, that the researcher will have a duty to inform health professionals if the participant discloses information which highlights any safeguarding or risk issues). All personal data will be kept in a locked filing cabinet in a locked office at the trial site and will be accessible only by researchers. Therapy files will be kept in a secure office and are not accessible to the staff collecting the research outcome data.

### Analysis

A sample size of n=20 per randomised group (n=40) will be sufficient to estimate a recruitment rate of 50% with a 95% CI of 34.51% to 65.9% and a retention rate of 80% with a 95% CI of 67.6% to 94.4% (PASS Software 2019). This sample size of 20 per arm will be sufficient to estimate the variability of the primary outcome measure (ie, changes in PSYRATS-AH and O-AS at post-treatment) for future sample size calculations, with 12 per arm sufficient for estimation of the variability for the purpose of sample size calculations.^47^ Difference between groups and 95% CI for the difference will be reported to aid sample size calculations for a definitive trial.

A complete statistical analysis will be drafted prior to recruitment beginning and finalised before any analysis is conducted. There will not be interim analyses or formal stopping rules in relation to this study as the primary goal is to establish feasibility parameters for a definitive trial. Data will be reported in accordance with the CONSORT principles, with the 2018 extension for reporting social and psychological intervention trials.^45^ We note recent CONSORT-SPI guidance, which recommends minimising the distinction between primary and secondary outcomes; therefore, all outcomes will be reported at the end of the trial.^48^ The feasibility data analysis and quantitative data analysis will be carried out by the MHRCT senior statistician clinical trials unit statistician and quality checked by a trial statistician.

Descriptive statistics will be used to summarise feasibility measures (ie, recruitment and retention, uptake of treatment, data completion) overall and by allocated group. Based on the ‘traffic light system/STOP-AMEND-GO’,^49^ progression criteria related to key feasibility outcomes such as recruitment, retention and treatment uptake rates have been proposed. Each criterion has ‘stop/amend/go’ indicators. For example, the progression criterion for treatment uptake will be 75% and above indicates the progression criterion has been met, 51–75% indicates the need to amend and below 50% indicates stop.

Clinical outcomes will be described using the mean (SD) or median (IQR) depending on the distribution of the measure and by the number (percentage) for binary outcomes. At baseline, participants will be reported overall and by randomised group. At follow-up points, participant outcomes will be presented by randomised group and the difference between groups and 95% CI for the difference will be reported to aid sample size calculations for the definitive trial. Outcomes will be reported for all randomised participants by randomised group, irrespective of whether they received the intervention or not (intention-to-treat). AEs will be reported for all randomised participants. Every effort will be made to follow-up with all participants for research assessments, and the analysis will use, where appropriate, statistical techniques for handling missing data. All analysis will be conducted in the latest version of R statistical package.

### Patient and public involvement

The LEAP at the Clinical Psychology and Digital Psychotherapy Department (MHRTC) was convened following the award of the grant supporting the present study. They have been invited to review the protocol, the choice of outcome measures, intervention procedures, recruitment methods and study materials, qualitative interview (format, content and data analysis), and dissemination of research findings. The LEAP has been built to advise on all the stages of the trial.

## Ethics and dissemination

The trial will be conducted in compliance with the principles of the Declaration of Helsinki,^50^ the principles of Good Clinical Practice and in accordance with all applicable regulatory requirements including but not limited to the Research Governance Framework and the Mental Capacity Act.^51^ It has received approval from the Ethics Committee of the Faculty of Psychology (R1/620). If amendments to the protocol are required, the same ethics committee will review them. The Ruhr Universität Bochum is the trial sponsor. The results of the study will be reported and disseminated at international conferences and in open-access peer-reviewed scientific journals. The results will also be available to participants and clinical teams in an accessible format and on the study website.

The study has been prospectively registered with the ISRCTN registry: ISRCTN 35980117. The study is monitored by the Ethics Commission at the Faculty of Psychology (Ruhr-Universität Bochum), and there is an independent Trial Steering Committee (TSC) and Live Experience Advisory Panel (LEAP) also supporting it.

## Trial status

Recruitment to the trial commenced in December 2023 (study protocol V.1.2, dated 24 September 2023) and is planned to finish in April 2025. Data collection is planned to continue until September 2026.
