# Supplementary material for: AVATAR Virtual Reality Social therapy (AVATAR_VRSocial) for distressing voices and their interference in social everyday life in early psychosis: protocol of a single-blind parallel group randomised controlled feasibility study
Source: BMJ Open. 2025 Apr 17;15(4):e098004. doi: 10.1136/bmjopen-2024-098004 (PMC12007037; doi:10.1136/bmjopen-2024-098004)
Supplement: Supplementary file 1 [file bmjopen-2024-098004.reviewer_comments.pdf]

PEER REVIEW HISTORY

BMJ Open publishes all reviews undertaken for accepted manuscripts. Reviewers are asked to complete a checklist review form and are provided with free text boxes to elaborate on their assessment. These free text comments are reproduced below.

ARTICLE DETAILS

Title (Provisional)

AVATAR Virtual Reality Social therapy (AVATAR_VRSocial) for distressing voices and their interference in social everyday life in early psychosis: protocol of a single-blind parallel group randomised controlled feasibility study.

Authors

Rus Calafell, Mar; Luker, Melina; Marzinzik, Maren; Nguyen, Phuong-Mi; Schneider, S.; Teismann, Tobias; Ehrbar, Nils; Tas, Ekincan; Zhang, Xiao Chi; Edwards, Clementine; Huckvale, Mark; Craig, Tom K J; Garety, Philippa; Ward, Thomas

---

VERSION 1 - REVIEW

**Reviewer**	**1**
**Name**	**Rosebrock, Laina**
**Affiliation**	**University of Oxford, Department of Experimental Psychology**
**Date**	**15-Jan-2025**
**COI**	**None**

This is an exciting and well thought out study with multiple components. The authors are taking an important and clear step forward in developing and enhancing treatments for individuals who hear distressing voices. I look forward to reading the results.

**Reviewer**	**2**
**Name**	**Veling, Wim**
**Affiliation**	**University of Groningen, Psychiatry**
**Date**	**28-Jan-2025**
**COI**	**None**

This project investigates the VR AVATAR therapy for people with first episode psychosis. The intervention has an additional part with VR social scenarios.

It would be helpful to strengthen the rationale for augmenting AVATAR therapy with VR social scenarios. Why would this be of added value? People often report that during social

interactions voices are in the background or absent. Is there any evidence that voice hearing plays a significant role during social interactions? Or that voice-related outcomes can be improved by practicing social scenarios?

This study adds VR social environments to AVATAR therapy, but the results will not inform us about the added value of the sessions with the VR environments. This is a key limitation. There is no comparison group of AVATAR without VR social scenarios, and there is no evaluation of the VR social scenarios part. The authors should explain how they intend to do this. The trial has already started, but I recommend to add at least an assessment of most important treatment outcomes between the regular AVATAR treatment sessions and the additional VR social scenarios sessions.

Exclusion criteria: what is the definition of “an acute mental health crisis”?

The CI has the right to withdraw participants from the study in the event of clinical contra-indications. What conditions would that be, and how are they assessed, reported and analyzed? We know from the Danish Challenge trial (VR AVATAR RCT) that many participants had increased symptoms after VR AVATAR sessions because of intensity of the VR therapy, and psychiatric hospital admissions were frequent.

VERSION 1 - AUTHOR RESPONSE

Reviewer: 1

Comments to the Author:

This is an exciting and well thought out study with multiple components. The authors are taking an important and clear step forward in developing and enhancing treatments for individuals who hear distressing voices. I look forward to reading the results.

Thank you for the comment and the encouragement. We are also looking forward to sharing our results with the wider clinical and research community.

Reviewer: 2

Comments to the Author:

This project investigates the VR AVATAR therapy for people with first episode psychosis. The intervention has an additional part with VR social scenarios.

It would be helpful to strengthen the rationale for augmenting AVATAR therapy with VR social scenarios. Why would this be of added value? People often report that during social interactions voices are in the background or absent. Is there any evidence that voice hearing plays a significant role during social interactions? Or that voice-related outcomes can be

improved by practicing social scenarios?

Thank you for this comment. Following the reviewer's comment, we have expanded the first paragraph of our introduction, where several relevant papers providing evidence about the relationship between hearing distressing voices and presenting difficulties during social interactions are summarised (page 4). The hypotheses around clinical outcomes cover both voice-related outcomes and social avoidance and distress. Two additional references have been added due to this change:

- Xu, Z., Mayer, B., Müller, M., Heekeren, K., Theodoridou, A., Dvorsky, D.,…Rüsch, N. (2016). Stigma and suicidal ideation among young people at risk of psychosis after one year. *Psychiatry Res*, *243*, 219-224. https://doi.org/10.1016/j.psychres.2016.06.041
- Sheaves, B., Johns, L., Černis, E., Griffith, L., Freeman, D., & McPin Hearing Voices Lived Experience Advisory Panel (2021). The challenges and opportunities of social connection when hearing derogatory and threatening voices: A thematic analysis with patients experiencing psychosis. *Psychol Psychother*, *94*(2), 341-356. https://doi.org/10.1111/papt.12303

This study adds VR social environments to AVATAR therapy, but the results will not inform us about the added value of the sessions with the VR environments. This is a key limitation. There is no comparison group of AVATAR without VR social scenarios, and there is no evaluation of the VR social scenarios part. The authors should explain how they intend to do this. The trial has already started, but I recommend to add at least an assessment of most important treatment outcomes between the regular AVATAR treatment sessions and the additional VR social scenarios sessions.

Thank you for this comment. The comparison of AVATAR_VRSocial with AVATAR therapy should be tested in a superiority trial, which requires a higher number of participants randomised to both conditions and appropriate specific analyses. Since the present study is also the first one testing AVATAR therapy exclusively in people at early stages of psychosis, and in a different national health system as the original works from the AVATAR core team (based in the United Kingdom), it appeared as a more rigorous step for the new augmented version to be tested first in a feasibility trial. We can assure the reviewer that this limitation will be stated clearly upon the publication of the results of the trial.

As specified in page 13, participants allocated to the experimental condition are assessed

weekly with the PSYRATS-AH. Following the reviewer's suggestion, we will also assess social anxiety and avoidance before starting the VR sessions. Although these measures will only be available for the experimental group, it will allow us to have some continuous data on the person session-by-session progress (page 13).

We strongly believe the qualitative interviews will also provide us with very valuable information about the additional value of the VR sessions, as well as perceived challenges and benefits of hearing the distressing voice when being in virtual social situations and interactions.

Exclusion criteria: what is the definition of "an acute mental health crisis"?

Thank you for this comment. The following clarification has been added (page 8):

Currently experiencing an acute mental health crisis (i.e., being at immediate and serious risk to self or others).

The CI has the right to withdraw participants from the study in the event of clinical contra-indications. What conditions would that be, and how are they assessed, reported and analyzed? We know from the Danish Challenge trial (VR AVATAR RCT) that many participants had increased symptoms after VR AVATAR sessions because of intensity of the VR therapy, and psychiatric hospital admissions were frequent.

Thank you for this comment. Following SPIRIT guidelines on recording of adverse events and study discontinuation, the following clarification has been added to the text (page 9):

The chief investigator (CI) also has the right to withdraw patients from the study in the event of clinical contra-indications: the intervention may be discontinued if the participant requests this or if the clinician judges, in consultation with the wider supervisory team, that the intervention is associated with a significant worsening of mental health. In this case an Adverse Event form would also be completed.

VERSION 2 - REVIEW

Reviewer 2

Name Veling, Wim

Affiliation University of Groningen, Psychiatry

Date 18-Mar-2025

COI

Thank you for your response to the points I raised.

Adding assessments of social anxiety and avoidance before starting the VR sessions will at least give an indication of the effectiveness of the AVATAR therapy without social scenarios.

I do not have any further questions, good luck with the study, I look forward to reading the results.
